# Bidirectional associations between sedentary time and sleep duration among 12- to 14-year-old adolescents

**DOI:** 10.1186/s12889-021-11694-9

**Published:** 2021-09-15

**Authors:** Maïté Verloigne, Veerle Van Oeckel, Ruben Brondeel, Louise Poppe

**Affiliations:** 1 Department of Public Health and Primary Care, Ghent University, Corneel Heymanslaan 10, 9000 Ghent, Belgium; 2 Department of Movement and Sports Sciences, Ghent University, Watersportlaan 10, 9000 Ghent, Belgium

**Keywords:** Sitting, Sedentary bouts, Prolonged sitting, Sleep behaviour, Sleep, Youth, Accelerometer, School

## Abstract

**Background:**

The aim of this study was to investigate bidirectional associations between (prolonged) sitting time and sleep duration in 12- to 14-year-old adolescents using a between-subjects and within-subjects analyses approach.

**Methods:**

Observational data were used from 108 adolescents (53% girls; mean age 12.9 (SD 0.7) years) from six schools in Flanders, Belgium. The Axivity AX3 triaxial accelerometer, worn on the thigh, was used to assess daily total sitting time and daily time spent in sedentary bouts of ≥30 min (as a proxy for prolonged sitting time). The Fitbit Charge 3 was used to assess nightly sleep duration. Both monitors were worn on schooldays only (ranging from 4 to 5 days). Linear mixed models were conducted to analyse the associations, resulting in four models. In each model, the independent variable (sleep duration, sitting time or prolonged sitting time) was included as within- as well as between-subjects factor.

**Results:**

Within-subjects analyses showed that when the adolescents sat more and when the adolescents spent more time sitting in bouts of ≥30 min than they usually did on a given day, they slept less during the following night (*p* = 0.01 and *p* = 0.05 (borderline significant), respectively). These associations were not significant in the other direction. Between-subjects analyses showed that adolescents who slept more on average, spent less time sitting (*p* = 0.006) and less time sitting in bouts of ≥30 min (*p* = 0.004) compared with adolescents who slept less on average. Conversely, adolescents who spent more time sitting on average and adolescents who spent more time sitting in bouts of ≥30 min on average, slept less (*p* = 0.02 and *p* = 0.003, respectively).

**Conclusions:**

Based on the between-subjects analyses, interventions focusing on reducing or regularly breaking up sitting time could improve adolescents’ sleep duration on a population level, and vice versa. However, the within-subjects association was only found in one direction and suggests that to sleep sufficiently during the night, adolescents might limit and regularly break up their sitting time the preceding day.

**Trial registration:**

Data have been used from our trial registered at ClinicalTrials.gov (NCT04327414; registered on March 11, 2020).

**Supplementary Information:**

The online version contains supplementary material available at 10.1186/s12889-021-11694-9.

## Background

Adolescents spend more than 60% of their daily waking hours sedentary [1]. Studies have found that higher levels of sedentary time are associated with more depressive symptoms, a less favourable weight status and decreased physical fitness among adolescents [2, 3]. Nevertheless, the evidence regarding the relation between sedentary time and health outcomes among adolescents is less consistent compared with adults [4, 5]. Next to the total volume of sedentary time per day, more prolonged sitting may also have a negative impact on adolescents’ health. Some studies found that more prolonged sitting is associated with increased cardiometabolic risk factors, a less favourable weight status and decreased physical fitness among youth [6, 7]; however, other studies found limited or no evidence for an association with health above and beyond the association of total sitting time [8, 9]. Nevertheless, sedentary behaviour guidelines across the world recommend to regularly break up prolonged sitting in addition to reducing the total sitting time [10].

Sleep behaviour has received increased attention in health promotion research as well. Although there are currently no international sleep guidelines, 14- to 17-year-old adolescents are recommended to sleep between 8 and 10 h per night and younger adolescents even between 9 and 11 h per night by paediatric sleep experts from the American National Sleep Foundation [11]. However, the proportion of adolescents meeting this recommendation ranges from 32% to 86% on schooldays, and 79% to 92% on non-schooldays [12]. In addition, these prevalence rates might even be an overestimation, as sleep onset latency (i.e., lying awake in bed before falling asleep) was not considered when calculating the sleep duration [13]. Insufficient sleep is associated with negative health outcomes in adolescents on the short and long term, including depressive symptoms, poor perceived mental health, increased likelihood of substance use [14, 15], and increased risk of developing cardiovascular disease, hypertension, type 2 diabetes and obesity [16].

Previous studies have examined the interrelatedness between both behaviours and suggest a bidirectional, negative association between sedentary time and sleep behaviour [17, 18]. These studies have applied and reported on a within-subjects analysis approach in order to reveal the acute effects of engaging more or less in a behaviour on a specific day or during a specific night [19]. Currently, there are two studies that have been conducted in adolescents. A study among 15-year-old adolescents found that on days with more sedentary time, adolescents had later sleep onset, shorter sleep duration, but higher sleep maintenance efficiency (i.e. the minutes of actual sleep between sleep onset and offset divided by the sleep duration interval, expressed in percentages) [20]. In the opposite direction, nights with longer sleep duration and later sleep offset, but also nights with a later sleep onset, predicted less sedentary time the day after. Another study in adolescents from the 6th and 8th grade also observed a bidirectional, negative association between sedentary time, screen-time and sleep duration, although the association varied by the specific sedentary and sleep health parameter used and by the time period of a day (e.g. sitting time during school hours vs. after school hours) [21]. In both studies sedentary time was measured using hip-worn accelerometers. Although hip-worn accelerometers are reliable and valid tools to measure adolescents’ physical (in)activity [22], they are unable to capture body posture [23]. Using physically inactive time as an indicator for sedentary time (i.e. time spent in a sitting position) might lead to mistakenly including standing [23, 24]. To more accurately assess sedentary time, it is highly recommended to use thigh-worn accelerometers [25]. Finally, no accelerometer-based studies among adolescents have examined the bidirectional association between prolonged sedentary time and sleep, although it is important to see whether the relation with sleep duration is different (e.g. stronger).

The aim of this study was to investigate the bidirectional association between objectively measured sedentary time and prolonged sedentary time with sleep duration in 12- to 14-year-old adolescents from Flanders, Belgium, using both a within- and between-subjects analysis approach. As mentioned before, the within-subjects analysis reveals the immediate effects of engaging more or less in a certain health behaviour, whereas the between-subjects analysis generates general knowledge on behavioural differences between individuals [19]. To unravel the association between the behaviours, it has been recommended to apply both approaches [26]. Our hypothesis was to find bidirectional, negative associations between (prolonged) sedentary time and sleep for the between- and within-subjects analyses.

## Methods

### Study protocol

For the purpose of this study, baseline data from a standing desks intervention study were analysed. A convenience sample of 22 secondary schools offering general secondary education located in East- and West-Flanders, Belgium, was invited to participate in this study. Twenty-two principals were contacted by email or phone, and six of them agreed to participate. In each school, one class of the 7th or 8th grade with a minimum of 20 pupils was selected by the principal. All children in those classes were invited to participate in the study (*n* = 132). In total, 123 children (response rate 93%) with written (parental) consent participated in the baseline measurement (February–March 2020). Measurements involved wearing two devices and completing a questionnaire (including sex and age). Due to COVID-19, the intervention and post-test measurement did not take place in Spring 2020. The study protocol was approved by the ethics committee of the Ghent University Hospital (B670201938818). The trial has been registered at ClinicalTrials.gov (NCT04327414; registered on March 11, 2020).

### Measurement of sedentary time variables and total volume of physical activity

#### Measurement protocol

To measure (prolonged) sitting time and total volume of physical activity (as a confounding factor), we used the Axivity AX3 triaxial accelerometer (Axivity, Newcaste, UK), a small waterproof device (dimensions: 23 × 32.5 × 7.6 mm; 11 g). Accelerometers were initialised to measure data at 25 Hz with 8G bandwidth. Initialising and downloading was executed via OmGui (version 1.0.0.43, GitHub, Inc., 2020). Adolescents were asked to continuously wear the device for one school week (4 or 5 school days), as the focus of the initial intervention study was on school day behaviour. Adolescents were asked to keep a diary on the time periods during which the device was not worn, if any. These periods of non-wear time were specified by noting the date and time of removal and the date and time of reattachment of the device. Devices were distributed at school on Monday or Tuesday and were collected again on Friday afternoon. The Axivity device was attached to the anterior mid-line of the adolescents’ right thigh using 3MTM Tegaderm Transparent Film Roll (3M, St. Paul, MN, USA). The device was oriented with the x-axis vertically (while standing) and the USB port facing down, y-axis horizontally to the left, and the z-axis horizontally forward.

#### Data processing

Data were loaded in R using the open-source software GGIR R package [27]. Non-wear time was detected using GGIR when on at least 1 axis, the standard deviation of the raw accelerometer data was lower than 13 m-gravitational unit (mg) and the range of the data was lower than 150 mg during 60 min blocks, evaluated per 15 min. In addition, when adolescents reported to have removed the Axivity in their diary, this was manually added as non-wear time during that period of time. Adolescents’ data on a daily basis were only included if a minimum of 9 h of accelerometer data during waking time was available. The waking time used while processing accelerometer data was defined as the time between sleep offset and sleep onset, measured by the Fitbit Charge 3 (see further in the Methods). In case an adolescent had partial missing Fitbit data, missing sleep offset and/or sleep onset was calculated based on the mean sleep offset and/or sleep onset of the other days. When no data regarding sleep offset or sleep onset were available, a fixed sleep offset (i.e. 7 AM) and onset (i.e. 10:30 PM) were used. This method of filling missing data was only used to determine waking time when processing the Axivity data, not to perform the statistical analyses in which sleep duration was used as a variable. In these analyses, missing Fitbit data were retained as missing data. Six types of activity were detected in 5 s bouts: (a) sitting, (b) lying, (c) standing, (d) walking, (e) cycling, and (f) running (expressed in min/day) [28]. Then, this variable was recoded as sedentary time (sitting and lying) and non-sedentary time. Total sedentary time per day was calculated by adding the 5 s bouts of sedentary time. The time spent in sedentary bouts of ≥30 min per day was used as a proxy of prolonged sitting; and calculated by adding the time spent in sedentary bouts of ≥30 min per day. A sedentary bout of ≥30 min was defined as a continuous period of sitting and/or lying time of at least 30 min, with no tolerance (i.e. interruption in the sitting and/or lying activity) allowed. To account for possible differences in wear time between adolescents, the two outcomes were expressed in percentage per day. To ensure the quality of the data, percentages exceeding 90 for sitting time and 81 (i.e. 90% of 90) for time spent in sedentary bouts of ≥30 min were removed from the analysis. The total volume of physical activity per day was measured as the mean Euclidean Norm Minus One (ENMO). The ENMO metric summarises the triaxial accelerometer data by taking the vector magnitude of the acceleration on the three axes at each observation (i.e. each 0.04 s); then extracting 1 g to accommodate for gravitation, rounding potential negative values to 0, and multiplying it by 1000 [29].

### Measurement of sleep duration

#### Measurement protocol

The Fitbit Charge 3 (Fitbit, Inc., San Francisco, CA, USA), a wrist-worn activity tracker including a triaxial accelerometer and heart rate monitor, was used to assess adolescents’ total sleep duration. Similar as to the Axivity, the Fitbit was continuously worn from Monday or Tuesday until Friday (also during the night). The psychometric properties of the Fitbit Charge 3 are not evaluated yet. However, two studies measured the performance of the Fitbit Charge HR in children and adolescents [30, 31]. The Fitbit Charge HR is the first Fitbit with multi-sensor capability, which is still used in its updated versions such as Charge 3. Both studies showed that the Fitbit Charge HR had an adequate sensitivity (respectively 97% and 95.7%) in detecting sleep against polysomnography. Nevertheless, the Fitbit Charge HR is not able to detect sleep stages, whereas more new-generation models (such as the Fitbit Charge 3) combine body movement and heart rate variability to identify and estimate time spent in sleep stages. Although only a very few studies have already evaluated the performance of the new-generation models (and not one of them evaluated the Fitbit Charge 3), it has been concluded that sleep-staging models perform better in assessing sleep behaviour [32].

#### Data processing

The Fitbit was synchronised using the Fitbit app (Fitbit, Inc., San Francisco, CA, USA), from which sleep onset and offset were extracted. Sleep onsets between 8 PM and 2 AM and sleep offsets between 5 AM and 9 AM were considered valid, which resulted in excluding 7 days from the analysis. Sleep duration was calculated as the time between sleep onset and offset.

### Statistical analyses

Considering the clustering of the data (i.e., days within participants), linear mixed models with two levels were used for analysing the data. The intraclass correlation (ICC) between observations from the same adolescents was calculated by running intercept-only models with each of the behavioural variables (i.e. sleep duration, percentage of time spent sitting and percentage of time spent sitting in bouts of ≥30 min) as outcome. The ICC provides information on the proportion of the total variance that is accounted for by grouping the data by participants. Consequently, the ICC reflects the correlation between observations belonging to the same participants.

To estimate the association between adolescents’ sleep duration and sedentary time during the following day, a model was fit with sedentary time as dependent variable and sleep duration on the previous night as between-subject (i.e. mean of the variable at the subject-level) as well as within-subject (i.e. subjects’ daily score minus their mean score) independent variables. The distinction between within- and between-subject variables was explicitly made because the standard mixed model approach does not make a distinction between both effects and implicitly assumes these effects are the same. However, incorrectly assuming similar effects can obscure the actual associations (see also Neuhaus and Kalbfleisch [33]). Similarly, for examining the association between sleep duration and prolonged sedentary time during the following day, a model was fit with prolonged sedentary time as dependent variable and sleep duration as between- as well as within-subject independent variables. In both models, adolescents’ age, sex and school were also included. To facilitate convergence, the variable ‘age’ was standardised before it was added to the model. To examine the association between adolescents’ sedentary time and sleep duration at the following night, a multilevel model was fit with sleep duration as dependent variable and sedentary time as between- as well as within-subject independent variables. Similarly, to investigate the association between prolonged sedentary time and sleep duration at the following night, a multilevel model was fit with sleep duration as dependent variable and prolonged sedentary time as between- and within-subject independent variables. In both models, adolescents’ age, sex, volume of total physical activity and school were also included. The variables ‘age’ and ‘total volume of physical activity’ were standardised when entered to the models. Finally, sensitivity analyses were performed to check the impact of deviations from participants’ average sleep duration at the night before. To do so, adolescents’ average sleep duration was subtracted from their sleep duration at the night before and entered into the model. For each of the fitted models, the assumptions were checked by visually inspecting the residuals versus fitted values plot and the quantile-quantile plot. *P*-values below 0.05 were considered statistically significant and below 0.1 borderline significant. The data were analysed using R version 4.0.3 (R Development Core Team, 2010), and the R-package lme4 version 1.1–25 [34]. A figure that visually represents the associations analysed in each model has been added as Additional File 1.

## Results

### Data availability

In total, 123 adolescents participated in the study. Two participants lost their Axivity accelerometer, nine participants did not wear a Fitbit during the night, one participant did not provide valid sleep data, two participants did not provide valid sedentary time data and one participant withdrew his/her consent. Hence, the analyses were performed on the data of 108 adolescents. The results of the models focusing on the association between (prolonged) sedentary time and sleep duration at the following night are based on 290 observations from 108 participants. The results of the models focusing on the association between sleep duration and (prolonged) sedentary time the following day are based on 182 observations from 105 participants.

### Descriptive statistics

Sample charachteristics are provided in Table 1. The median number of valid days (i.e. days with ≥9 h of wear time during waking time) of Axivity data was 4 out of a maximum of 5. The median number of nights during which participants correctly wore the Fitbit was 3 out of a maximum of 4. The ICC of 0.50 found for sleep time indicates that the differences between participants in sleep time were similar to the differences detected within participants. The ICCs for the percentage of waking time spent sitting and the percentage of waking time spent in bouts of ≥30 min are very similar, 0.28 and 0.27 respectively. These low ICCs indicate that the variation was mainly accounted for by differences within individuals. Hence, within participants, there was substantial variation in the time spent sedentary (in bouts of ≥30 min) from day to day.

**Table 1:** Characteristics of the sample

Characteristics	N (%) / Mean	SD	Range	ICC
Sex				
Girls	57 (53)			
Boys	51 (47)			
Age in years	12.91	0.67	12.00–15.00	
Sleep time in hours	8.37	0.67	4.76–9.88	0.50
Percentage of waking time spent sitting	71.34	5.50	48.53–89.98	0.28
Percentage of waking time spent sitting in bouts of ≥30 min	36.54	9.12	8.80–75.32	0.27

*SD* Standard deviation, *ICC* Intraclass correlation between observations within the same adolescents

### The association between sedentary time and sleep duration at the following night

Table 2 presents the results of the model examining the association between the percentage of waking time spent sitting and sleep duration at the following night. This model did not adequately converge when the variable “school” was included. However, because similar results were obtained by removing school from the model, we decided to report the results of the original model. The significant within-subject association between sedentary time and sleep duration at the following night (beta(SE) = − 0.02(0.01); *p* = 0.01) indicates that, when the adolescents sat more than they usually did on a given day, they slept less during the following night. This association was independent of their total volume of physical activity that day and their average sedentary time. Furthermore, a significant between-subject association between sedentary time and sleep duration the following night was detected (beta(SE) = − 0.03(0.01); *p* = 0.02), indicating that adolescents who spent more time sitting on average slept less compared with adolescents who spent less time sitting on average. Similar results were obtained with the sensitivity analysis including adolescents’ deviation in sleep duration at the night before as predictor (see Additional file 2).

**Table 2:** Results of the model examining the association between (prolonged) sedentary time and sleep duration at the following night

	Sleep duration	
Independent variables	Regression coefficient	95% confidence interval
(Intercept)	10.48	8.76; 12.20***
Sedentary time (within-subject)	−0.02	− 0.04; − 0.005*
Sedentary time (between-subject)	−0.03	− 0.06; − 0.005*
(Intercept)	9.10	8.54; 9.65***
Prolonged sedentary time (within-subject)	−0.01	−0.02;-0.0002˟
Prolonged sedentary time (between-subject)	−0.02	−0.04;-0.008**

***p < .001 **p < .01 *p < .05 ˟*p* < .10; Controlled for age, sex, total volume of physical activity, and school

### The association between prolonged sedentary time and sleep duration at the following night

A borderline significant within-subject association between prolonged sedentary time and sleep duration at the following night was identified (beta(SE) = − 0.01(0.004); *p* = 0.05; see Table 2). This indicates that, when adolescents spent more time sitting in bouts of ≥30 min, they slept less during the following night. This borderline significant association was independent from their level of physical activity and their average sitting time. Furthermore, adolescents who spent on average more time sitting in bouts of ≥30 min slept less than adolescents who spent on average less time sitting in bouts of ≥30 min (beta(SE) = − 0.02(0.01); *p* = 0.003). Similar results were obtained with the sensitivity analysis including adolescents’ deviation in sleep duration at the night before as predictor (see Additional file 2).

### The association between sleep duration and sedentary time the following day

A significant association between adolescents’ mean sleep duration and sitting time was detected (beta(SE) = −2.30(0.83); *p* = 0.006; see Table 3), indicating that adolescents who slept more on average spent less time sitting than adolescents who slept less on average.

**Table 3:** Results of the model examining the association between sleep duration and (prolonged) sedentary time the following day

	Sedentary time		Prolonged sedentary time	
Independent variables	Regression coefficient	95% confidence interval	Regression coefficient	95% confidence interval
(Intercept)	86.00	72.32; 99.67***	69.08	43.35; 95.34***
Sleep duration (within-subject)	0.57	−1.60; 2.74	−0.09	−3.83; 3.74
Sleep duration (between-subject)	−2.30	−3.88; −0.72**	− 4.60	− 7.64; − 1.63**

***p < .001 **p < .01; Controlled for age, sex, and school

### The association between sleep duration and prolonged sedentary time the following day

A significant between-subject association between sleep duration and prolonged sedentary time was detected (beta(SE) = − 4.60(1.58); *p* = 0.004; see Table 3), indicating that adolescents who slept more on average spent less time sitting in bouts of ≥30 min than adolescents who slept less on average.

## Discussion

The aim of this study was to investigate bidirectional associations between (prolonged) sedentary time and sleep duration in 12- to 14-year old adolescents from Flanders, Belgium. Within-subject results showed that adolescents who sit more than they normally do on a specific school day, sleep less the following night. In addition, adolescents who engage in more prolonged sitting than they normally do on a specific school day, also sleep less the following night, although the association was only borderline significant.

Although various mechanisms to explain the acute effect of physical activity on sleep have already been suggested [35], there is not much evidence on the mechanisms explaining the acute effect of sitting on sleep. Therefore, we suggest some hypotheses below, but future research should verify them. A first possible explanation could be that specific factors mediate the association between more (prolonged) sitting and less sleep. For example, high levels of sedentary time and engaging in long sedentary bouts are related to negative emotions in adolescents, such as feeling unwell or stress [36]. Experiencing negative emotions has in turn an impact on adolescents’ sleep onset, resulting in a short sleep duration [37]. Another example is that more sitting and more prolonged sitting (i.e. less breaks in sitting time) on a given day are both associated with less energy expenditure [38]. This could result in feeling less sleepy in the evening and not feeling the urge to go to bed [20]. This might especially be detrimental for adolescents, as biologically they already have an evening circadian phase preference for later bed times [39]. So in terms of sleeping sufficiently during the night, an important and useful strategy for adolescents might thus be to not only limit but also to break up the time spent sedentary the preceding day. Another explanation for the acute effect could be that the additional sedentary time of the adolescents on a specific day is spent in the form of screen-time activities during the evening period [40]. This would imply that it is not the sitting behaviour itself that is important for sleeping sufficiently the following night, but rather the activity that is done while sitting. Indeed, screen-time activities are an important source of entertainment and might therefore push adolescents to postpone their bed time [41]. This results in a shorter sleep duration, as rise time is mostly fixed on schooldays. Additionally, such screen-time activities can involve long bouts of sedentary time, such as binge watching, which explains the specific association between prolonged sitting and sleep duration. Extra analyses conducted as part of this study confirm that it is the additional (prolonged) sedentary time during non-school hours that is significantly related to a shorter sleep duration, and not the additional (prolonged) sedentary time during school hours (see Additional file 3). This is in line with the study of Kim and colleagues [21] in which more sedentary time during the after-school period, and not during the school period, was associated with a shorter sleep duration the following night among young adolescents. This means that when promoting the reduction and breaking up of sedentary time during the day, there should be a focus on the non-school hours.

Unexpectedly, the association was not found in the other direction: sleep duration during the night was not significantly related to more sedentary time or to more time in long sedentary bouts on the following day. However, other studies found the association in both directions (e.g. [17, 20]), suggesting our results do not completely align. A possible reason why sleep on a given night did not affect sedentary time the next day in our study, might be that we only collected data on school days. Previous research has attributed some of the within-variability among adolescents to the different sleeping patterns between weekdays and weekend days (i.e. catching up the loss of sleep from the school week during the weekend) [42], suggesting that there might be less within-variability between the different weekdays. In addition, our study sample included relatively young adolescents, and adolescents’ age has shown to be a positive predictor of the within-variability in total sleep duration [42, 43]. However, one of the studies that found an association between sleep duration on a given night and sedentary time the following day also only included school days in young adolescents, suggesting other factors might also be relevant to explain the difference between study findings. The difference in measurement method could play a role here, as previous studies have used hip-worn accelerometers to measure both sedentary time as well as sleep [17, 20]. However, hip-worn accelerometers cannot accurately distinguish standing from sitting [23, 24], suggesting our estimates of time spent sitting are more accurate than the estimates of other studies. For sleep, it is less clear if hip- or wrist-worn devices are preferred, although sleep has been validated mainly with wrist-worn devices [44]. Hip-worn devices have been shown to overestimate sleep duration compared to wrist-worn devices [45], however, others have stated that hip-worn devices are superior to assess sleep duration [46].

In contrast to the within-subjects results, the between-subjects analyses revealed a bidirectional association. Adolescents who, on average, sleep less, have higher levels of sedentary time and spend more time in prolonged sitting. It was also found that adolescents who, on average, have higher levels of sedentary time or spend more time in prolonged sitting, have a shorter sleep duration. These two modifiable health behaviours are thus in general interrelated, which means future interventions focusing on reducing or regularly breaking up sedentary time could improve adolescents’ sleeping behaviour on a population level, and vice versa. This can be important for age-specific obesity prevention programmes, as sedentary time and sleep have been identified as two factors influencing its development [2, 16]. This would imply that the health effect of population-based interventions focusing on one behaviour (sedentary time or sleep) might be reinforced because of a concurrent effect on the other behaviour. However, it must be acknowledged that effect sizes were relatively small.

A first strength of this study is that both within-subjects as between-subjects analyses were conducted in order to unravel the association between sleep and sedentary time in adolescents. Between-subjects analyses inform us about inter-individual differences or in other words, the general interrelatedness of sedentary time and sleep duration (i.e. on a trait level), whereas within-subjects analyses inform us about intra-individual differences or the immediate effects of sitting/sleeping more or less than usual (i.e. on a state level) [19]. Another strength is the inclusion of spending time in sedentary bouts of at least 30 min as a proxy for prolonged sitting on a day. Previous studies have only investigated relationships between levels of sedentary time and sleep [17, 20, 21], and it has been advocated to examine the associations between breaks in sedentary time and sleep as well [26]. The current study adds to the literature by showing that not only the total time spent sedentary, but also how it is accumulated is important for adolescents’ sleep. A final strength is the use of device-based instruments to objectively measure sitting time, the time spent sitting in bouts of at least 30 min and sleep duration. Moreover, we used a thigh-worn accelerometer, which is more accurate to estimate people’s sedentary time compared to a hip-worn accelerometer [25]. However, the disadvantage is that there is no information on the specific sedentary activities performed, suggesting future research should combine different measurement methods. Study limitations include the convenience sampling method and specific focus on secondary schools offering general education, limiting the representativeness of our findings. Another limitation is that we only had data on school days because of the nature of the larger school-based intervention study from which data were used. A final limitation is that we did not have day-to-day data on adolescents’ sleep quality. Although sleep duration and sleep quality are inherently associated with each other, it has been advocated that sleep quality is the most important health parameter [47, 48]. Future studies could thus use ecological momentary assessment methods to assess adolescents’ sleep quality.

## Conclusions

The between-subjects analyses in this study showed a bidirectional, negative association between (prolonged) sedentary time and sleep duration on weekdays in 12- to 14-year-old adolescents, suggesting population-based interventions focusing on a general improvement of one behaviour might affect the other health behaviour. However, the within-subjects analyses only revealed a negative association between (prolonged) sedentary time and sleep duration, but not in the other direction. This suggests that more sitting and more prolonged sitting than normally on a weekday has an immediate, negative effect on the sleep duration the following night. This might be an important public health message for an adolescent population. Future research could disentangle the mechanisms underlying these associations.

## Supplementary Information

**Additional file 1:** A visual representation of the four statistical models.

**Additional file 2:** Results of the sensitivity analysis examining the association between (prolonged) sedentary time and sleep duration at the following night.

**Additional file 3:** Results of the models examining the association between (prolonged) sedentary time at school/elsewhere and sleep duration at the following night.

## Data Availability

The datasets and R-script used and/or analysed during the current study are available from the corresponding author on reasonable request.

## Abbreviations

ENMO: Euclidean Norm Minus One
SD: Standard deviation
SE: Standard error
ICC: Intraclass correlation between observations within the same adolescents
