# Supplementary figures and images for: Bidirectional associations between sedentary time and sleep duration among 12- to 14-year-old adolescents
Source: BMC Public Health. 2021 Sep 15;21:1673. doi: 10.1186/s12889-021-11694-9 (PMC8440143; doi:10.1186/s12889-021-11694-9)

Figure 1: A visual representation of the four statistical models


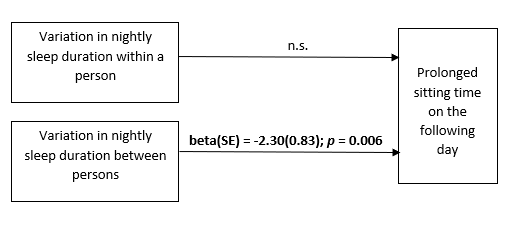

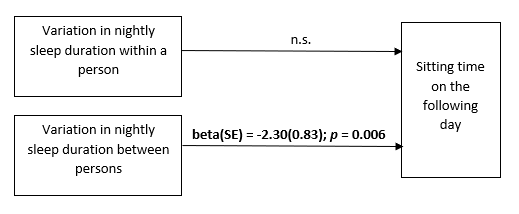

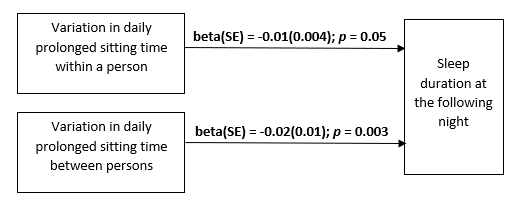

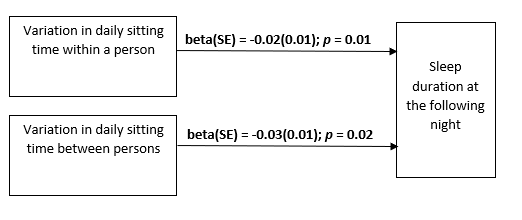


n.s., not significant

Supplement: Supplementary file 1 — Additional file 1: A visual representation of the four statistical models. [file 12889_2021_11694_MOESM1_ESM.docx]
