# Supplementary material for: Bidirectional associations between sedentary time and sleep duration among 12- to 14-year-old adolescents
Source: BMC Public Health. 2021 Sep 15;21:1673. doi: 10.1186/s12889-021-11694-9 (PMC8440143; doi:10.1186/s12889-021-11694-9)
Supplement: Supplementary file 2 — Additional file 2: Results of the sensitivity analysis examining the association between (prolonged) sedentary time and sleep duration at the following night. [file 12889_2021_11694_MOESM2_ESM.docx]

Sensitivity analyses

*The association between sitting time and sleep duration at the following night*

Supplementary table 1. Results of the sensitivity analysis examining the association between sitting time and sleep duration at the following night

| **Independent variables** | **Regression coefficient** | **95% confidence interval** |
| --- | --- | --- |
| (Intercept) | 10.21 | 8.48; 11.94*** |
| Sitting time (within-subject) | -0.02 | -0.04; -0.006* |
| Sitting time (between-subject) | -0.03 | -0.05; -0.0002˟ |

Notes. *** *p*<.001 ***p*<.01 **p*<.05 ˟*p*<.10; Controlled for age, sex, total volume of PA, school, and deviation from sleep time on the day before.

*The association between prolonged sitting time and sleep duration at the following night*

Supplementary table 2. Results of the sensitivity analysis examining the association between prolonged sitting time and sleep duration at the following night

| **Independent variables** | **Regression coefficient** | **95% confidence interval** |
| --- | --- | --- |
| (Intercept) | 8.98 | 8.43; 9.53*** |
| Prolonged sitting time (within-subject) | -0.01 | -0.02; -0.003* |
| Prolonged sitting time (between-subject) | -0.02 | -0.03; -0.003* |

Notes. *** *p*<.001 ***p*<.01 **p*<.05 ˟*p*<.10; Controlled for age, sex, total volume of PA, school, and deviation from sleep time on the day before.
