# Supplementary material for: Bidirectional associations between sedentary time and sleep duration among 12- to 14-year-old adolescents
Source: BMC Public Health. 2021 Sep 15;21:1673. doi: 10.1186/s12889-021-11694-9 (PMC8440143; doi:10.1186/s12889-021-11694-9)
Supplement: Supplementary file 3 — Additional file 3: Results of the models examining the association between (prolonged) sedentary time at school/elsewhere and sleep duration at the following night. [file 12889_2021_11694_MOESM3_ESM.docx]

Association between (prolonged) sitting time at school/elsewhere and sleep duration

Supplementary table 3. Results of the models examining the association between sitting time at school/elsewhere and sleep duration at the following night.

|  | **At school** | | **Elsewhere** | |
| --- | --- | --- | --- | --- |
| **Independent variables** | **Regression coefficient** | **95% confidence interval** | **Regression coefficient** | **95% confidence interval** |
| (Intercept) | 8.70 | 6.48; 10.93*** | 10.62 | 9.36; 11.88*** |
| Sitting time  (within-subject) | 0.001 | -0.01; 0.02 | -0.01 | -0.02; -0.0002˟ |
| Sitting time  (between-subject) | -0.003 | -0.04; 0.03 | -0.03 | -0.05; -0.01** |

Supplementary table 4. Results of the models examining the association between prolonged sitting time at school/elsewhere and sleep duration at the following night.

|  | **At school** | | **Elsewhere** | |
| --- | --- | --- | --- | --- |
| **Independent variables** | **Regression coefficient** | **95% confidence interval** | **Regression coefficient** | **95% confidence interval** |
| (Intercept) | 8.60 | 8.06; 9.14*** | 9.20 | 8.78; 9.62*** |
| Prolonged sitting time  (within-subject) | 0.004 | -0.003; 0.01 | -0.008 | -0.01; -0.003** |
| Prolonged sitting time  (between-subject) | -0.003 | -0.01; 0.01 | -0.03 | -0.04; -0.02*** |
